# Efficacy of repeated immunoadsorption in patients with post-COVID myalgic encephalomyelitis/chronic fatigue syndrome and elevated β2-adrenergic receptor autoantibodies: a prospective cohort study

**DOI:** 10.1016/j.lanepe.2024.101161

**Published:** 2024-12-12

**Authors:** Elisa Stein, Cornelia Heindrich, Kirsten Wittke, Claudia Kedor, Rebekka Rust, Helma Freitag, Franziska Sotzny, Anne Krüger, Markus Tölle, Patricia Grabowski, Carmen Scheibenbogen, Laura Kim

**Affiliations:** a Institute of Medical Immunology, Charité - Universitätsmedizin Berlin, Corporate Member of Freie Universität Berlin and Humboldt-Universität zu Berlin, Augustenburger Platz 1, Berlin, 13353, Germany; b Department of Nephrology, Charité - Universitätsmedizin Berlin, Corporate Member of Freie Universität Berlin and Humboldt-Universität zu Berlin, Augustenburger Platz 1, Berlin, 13353, Germany; c Experimental and Clinical Research Center and Neuro Science Clinical Research Center, Charité - Universitätsmedizin Berlin, Corporate Member of Freie Universität Berlin, Humboldt Universität zu Berlin, Berlin Institute of Health, Charitéplatz 1, Berlin, 10117, Germany

**Keywords:** Myalgic encephalomyelitis, Chronic fatigue syndrome, COVID-19, SARS-CoV-2, Pandemic, Post-COVID syndrome, Immunoadsorption, Plasmapheresis, Autoantibodies

## Abstract

**Background:**

Since the pandemic, severe acute respiratory syndrome coronavirus 2 (SARS-CoV-2) has become the leading trigger for myalgic encephalomyelitis/chronic fatigue syndrome (ME/CFS). Evidence indicates that autoimmunity plays an important pathophysiological role. We aimed to evaluate the effectiveness of IA treatment in post-COVID ME/CFS patients.

**Methods:**

This pre-post study included 20 post-coronavirus disease 2019 (COVID) ME/CFS patients found to have elevated β2 adrenergic autoantibodies (β2 AR-AB) between October 2022 and October 2023. Patients, with a median disease duration of 22 months (IQR: 15–31), were treated with five immunoadsorption sessions at Charité - Universitätsmedizin Berlin, Germany. Seven were male and 13 female, with a median age of 40 years (IQR: 36–51). The primary end point was the change in the Short Form (36) Health Survey physical functioning domain (SF36 PF) from baseline to four weeks post immunoadsorption. Key symptoms were assessed via questionnaires over six months. Handgrip strength and EndoPAT® measurements were used to evaluate muscle fatigue and vascular dysfunction. Seven patients who worsened after an initial response received a second cycle.

**Findings:**

The treatment was generally well tolerated, reducing total immunoglobulin G by 79% (*CI*: 73–84%) and β2 AR-AB by 77% (*CI*: 58–95%). Patients demonstrated a mean increase in the SF36 PF of 17.75 points (*CI*: 13.41–26.16), with the greatest improvement occurring between months two and three, and significant gains maintained through month six. 14/20 (70%) patients were categorized as responders with an increase in the SF36 PF of ≥ ten points. Further lasting improvements were reported in fatigue, post-exertional malaise, pain, cognitive, autonomic, and immunological symptoms. Female patients had increased repeat handgrip strength at month six.

**Interpretation:**

Immunoadsorption may improve symptoms in post-COVID ME/CFS patients. The beneficial effects of IgG depletion suggest a significant role for autoantibodies and disturbed B-cell function in the condition's pathophysiology.

**Funding:**

Funded by The 10.13039/501100002347Federal Ministry of Education and Research and the Weidenhammer Zöbele Research Foundation.

**Research in context**

**Evidence before this study**

We searched PubMed for relevant studies published from March 2020 to May 2024, focusing on the COVID-19 pandemic. Search terms included “Post-Acute COVID-19 Syndrome” [MeSH Terms] OR “Fatigue Syndrome, Chronic” [MeSH Terms] AND “Drug Therapy” [MeSH Subheading]. Since the pathomechanisms remain largely uncertain, no mechanistic or causal therapy has been identified for either ME/CFS or post-COVID syndrome. Among the results, only a few randomized controlled trials (RCTs) were found, primarily assessing supplements as symptomatic therapy. Besides supplements, current treatment approaches under evaluation in observational trials include antiviral agents such as nirmatrelvir + ritonavir, anticoagulants, and drug repurposing of anti-inflammatory, vasodilators, psychiatric medications or low-dose naltrexone. We have already conducted two small proof-of-concept studies on IgG depletion via immunoadsorption (IA) in pre-pandemic post-infectious ME/CFS patients, which showed efficacy in most patients. IA has therefore been suggested as a potentially feasible approach for post-COVID patients by other research groups; however, no other trials on this subject have been completed yet.

**Added value of this study**

Our study is the first to demonstrate, that IA treatment can significantly alleviate a range of debilitating symptoms in most post-COVID ME/CFS patients, such as fatigue, pain, and cognitive as well as autonomic dysfunction. Remarkably, these clinical improvements were observed as early as four weeks post-treatment and lasted up to six months. This not only suggests a significant role for autoantibodies and disturbed B-cell function in the pathophysiology of post-COVID ME/CFS but also has implications for future therapeutic approaches.

**Implications of all the available evidence**

In summary, our data suggest that IA treatment may result in rapid clinical improvement in a subset of patients. Our findings lay the groundwork for consecutive clinical trials, a randomized controlled trial (RCT) with sham apheresis, and combining IA with subsequent B-cell depletion.

## Introduction

Myalgic encephalomyelitis/chronic fatigue syndrome (ME/CFS) is an acquired multi-system disease characterized by persistent fatigue and exertional intolerance with a disproportionate worsening after physical or cognitive exertion referred to as post-exertional malaise (PEM). Furthermore, it is accompanied by a variety of other symptoms that are related to immunological, cognitive, and autonomic dysfunction.

The worldwide prevalence was estimated at 0.89% in a pre-pandemic meta-analysis, though estimates vary according to the case definitions used.^1^ While there is evidence for a genetic predisposition to ME/CFS,^2^ it is most often triggered by viral infections.^3^ Since the COVID-19 pandemic, SARS-CoV-2 has become the leading viral trigger for ME/CFS.^4^ Given the high incidence of COVID-19, the prevalence of ME/CFS is expected to strongly increase.

The diagnosis of ME/CFS is based on established diagnostic criteria, with the most frequently used being the Canadian Consensus Criteria (CCC) and the Institute of Medicine (IOM) criteria. These require the presence of key symptoms including lingering fatigue, PEM, unrefreshing sleep, cognitive impairment, and/or orthostatic intolerance.^5^ ME/CFS diagnosis relies so far on these clinical criteria, although most patients present with objective clinical findings including diminished handgrip strength or autonomic dysfunction.^4^

The pathophysiological mechanism of ME/CFS is not yet known, but numerous studies suggest dysregulation of the immune system. Elevated antibodies were found in ME/CFS patients in several studies, most frequently against adrenergic and muscarinic receptors.^6^^,^^7^ Elevated β1 and β2 adrenergic receptor autoantibodies (β1/β2 AR-AB) and M3/M4 acetylcholine receptor autoantibodies (M3/M4 AChR-AB) are likely to modulate the autonomic nervous system function and vasoregulation.^7^^,^^8^

Levels of these autoantibodies were found to be associated with symptom severity and structural alterations in the central nervous system in both post-COVID syndrome (PCS) and ME/CFS.^9^, ^10^, ^11^, ^12^, ^13^ The β2 AR-AB was the best marker for distinguishing PCS from recovered patients, and levels of β2 AR-AB were associated with both fatigue and vasomotor symptoms in PCS-ME/CFS patients.^10^^,^^14^ Apart from elevated autoantibodies a chronic activation of inflammatory pathways and an alteration in memory B-cells were shown.^15^^,^^16^

The goal of immunoadsorption (IA) is to improve the clinical condition of patients with autoantibody-mediated diseases by selectively removing immunoglobulins from circulation via extracorporeal adsorption from their plasma.^17^^,^^18^ Unlike plasmapheresis, IA employs a high-affinity column that specifically binds and eliminates immunoglobulins including autoantibodies and immune complexes, leaving other plasma components behind.^18^

Given the previous evidence for a potential role of autoantibodies in PCS and ME/CFS and the effectiveness of IA in postinfectious ME/CFS, we here aimed to evaluate the effectiveness of IA treatment specifically in post-COVID ME/CFS patients.^19^^,^^20^ Effectiveness was measured by assessing patient-reported outcomes and hand grip strength. We hypothesized that IA would lead to clinical improvement in ME/CFS patients four weeks after treatment, with an additional beneficial effect from repeat IA in responders with relapse.

## Methods

### Study design

This prospective cohort study, conducted at the outpatient department for immunodeficiencies at the Institute of Medical Immunology, Charité - Universitätsmedizin Berlin, recruited patients between October 2022 and October 2023. Patients received IA treatment and follow-up visits for 12 months. Responders to the first cycle of IA were offered a second cycle. An interim report of the first 10 patients was published in October 2023.^21^ This study was being conducted within the National Clinical Studies Group (NKSG), a clinical trial and translational research platform focused on developing therapies for PCS and ME/CFS, funded by the German Ministry of Education and Research (BMBF).^22^

### Patients

Patients were diagnosed and recruited at the outpatient department for immunodeficiencies at the Institute of Medical Immunology at the Charité - Universitätsmedizin Berlin. The diagnosis of ME/CFS was based on the modified Canadian Consensus Criteria (CCC) and a minimum of 14 h of PEM.^23^^,^^24^ Inclusion criteria encompassed elevated β2 AR-AB at the time of screening and SARS-CoV-2 infection at the time of disease onset. All patients had to provide proof of SARS-CoV-2 infection by positive PCR, antigen test, or serology (SARS-CoV-2 nucleocapsid protein antibodies).

Patients were excluded from this study if they had relevant comorbidities,^25^ pre-existing fatigue, evidence of organ dysfunction, or acute or chronic infections such as HIV or hepatitis. Additionally, patients who were unable to leave their homes due to the severity of their illness were also excluded.

All patients signed informed consent before study inclusion. The Ethics Committee of the Charité - Universitätsmedizin Berlin approved this study in accordance with the 1964 Declaration of Helsinki and its later amendments (protocol code EA2/134/22, date of approval: 28 July 2022).

### Procedures

Five sessions of IA were administered at the Department of Nephrology at Charité - Universitätsmedizin Berlin. IA treatment was conducted in an outpatient setting over a ten-day period, with sessions spaced no more than two days apart. The TheraSorb® – Ig omni 5 adsorber (Miltenyi Biotech B.V. & Co. KG, Bergisch Gladbach, Germany) was used for removal of human lambda and kappa chains containing immunoglobulins IgG (subclasses IgG1-IgG4), IgA, IgM, IgE, and immune complexes as well as free lambda and kappa light chains from the plasma.

To ascertain the efficacy of IA, total serum immunoglobulin levels were assessed via immunoturbidimetry before, during, and after treatment. Antibodies against β1, β2, M3, and M4 receptors were determined using ELISA technology by CellTrend GmbH, Luckenwalde, Germany, both before and after treatment. Intra- and inter-assay coefficients of variation for the ELISAs provided by CellTrend were: b1 AR-AB 9.6%/12.0%, b2 AR-AB 4.2%/3.8%, M3 AchR-AB 5.9%/10.1%, and M4 AchR-AB 7.3%/12.5%. Pre- and post-treatment samples were analyzed in the same assay run. The upper normal levels of autoantibodies were determined based on validation studies of a healthy control group and defined as being larger than the 90th percentile of a healthy control group (>14U/l for β2 AR-AB).

For patient-reported outcomes, questionnaires were filled out before, during, and after the treatment in monthly intervals and validated by physicians. Patients’ health-related quality of life was assessed using the 36-Item Short-Form Survey (SF-36), with scores ranging from 0 to 100, with 100 indicating no limitations.^26^ Response to IA treatment was defined as a minimum increase in the SF-36 physical functioning domain (SF-36 PF) of 10 points from baseline to four weeks post IA, indicating a clinically relevant improvement.^27^ Fatigue was evaluated using the Fatigue Severity Scale (FSS), ranging from 9 to 63, with a total score of 36 or more suggesting relevant fatigue.^28^ Additionally, disease-related disability was scored according to the Bell score, rating the restriction in daily functioning on a scale from 0 to 100, with 100 indicating no restriction.^29^ PEM was evaluated using the DePaul Symptom Questionnaire (PEM-DSQ), ranging from 0 to 20 for both severity and frequency, PEM duration was assessed ranging from 0 to 6, with higher values indicating higher PEM severity.^24^ Further cardinal symptoms of both PCS and ME/CFS, including fatigue, muscle pain, immunological symptoms, and cognitive impairment, were scored on a numeric rating scale (NRS) from 1 to 10, with 10 indicating maximum symptom severity (not formally validated). Autonomic dysfunction was evaluated according to the Composite Autonomic Symptom Score (COMPASS31), ranging from 0 to 100, with 100 indicating maximum autonomic dysfunction.^30^

Handgrip strength (HGS) of the dominant hand was measured using a digital hand dynamometer (EH101, Deyard, Shenzhen, China) in two separate sessions. Rest time between sessions was 60 min, in which no strenuous physical activity took place. Before starting the measurement, patients were shown two separate demonstrations of how the dynamometer should be used. Patients sat in an upright position facing a standard table during measurements of HGS. The forearm of the dominant hand was placed on the table in full supination holding the dynamometer. Under supervision and verbal motivation, the handle was pulled 10 times with maximum force for three seconds, followed by a five-second relaxation phase. The dynamometer displayed the highest value reached within these three seconds (measurement in kg), this single value was then recorded. The attempt with the highest reading out of ten repetitions was recorded as the maximum strength (Fmax), and the average strength (Fmean) of each session was calculated.^31^

Further, the Reactive hyperemia index (RHI), which is a measure for endothelial function, was assessed using a peripheral arterial tonometry device (EndoPAT 2000, Itamar Medical, Israel). The technology measures the pulsatile volume changes in the vascular beds of the finger using optical sensors. The subjects were in supine position for a minimum of 15 min before measurements, in a quiet, temperature-controlled room. Occlusion of the brachial artery was performed on the nondominant upper arm using a standard blood pressure cuff. The occlusion pressure was at least 60 mmHg above the systolic blood pressure. Upon release of the cuff, the resulting surge in blood flow causes vessel dilation. Each recording consisted of five minutes of baseline measurement, five minutes of occlusion measurement, and five minutes post-occlusion measurement. The post-occlusion dilation relative to pre-occlusion levels is calculated as the RHI. Endothelial dysfunction was defined as an RHI ≤ 1.67 based on previous cohort studies.^32^

Study data were collected and managed using the REDCap electronic data capture tools hosted at the Charité - Universitätsmedizin Berlin.^33^^,^^34^

### Statistical analysis

Statistical analyses were conducted using R version 4.3.0 and RStudio version 2023.03.1. A linear mixed-effects model (LMM) was employed to assess changes in multiple outcome variables across different time points. The analysis was performed using the lmer function from the lme4 package (version 1.1-35.5), and ggplot2 (version 3.5.0) was utilized for data visualization. For each outcome variable, the LMM included time as a fixed effect and patient number as a random effect to account for within-patient correlation. The mixed model was fitted using restricted maximum likelihood (REML), and statistical significance was evaluated using t-tests with *p*-values approximated through Satterthwaite's method for degrees of freedom, implemented via the lmerTest package (version 3.1-3). Missing data were accounted for by using all available observations in the model, allowing for the estimation of fixed and random effects without listwise deletion, assuming data are missing at random.

For comparisons between groups the non-parametric Whitney-U test was used. Correlation analysis was performed using the nonparametric Spearman coefficient. A two-tailed *p*-value of <0.05 was considered to provide evidence of a statistically significant result.

### Role of the funding source

The funder of the study had no role in study design, data collection, data analysis, data interpretation, or writing of the report.

## Results

The β2 AR-AB levels of 402 patients, who met the CCC for post-infectious ME/CFS, were measured between June 2022 and March 2023. Out of these 402 patients, 189 (47%) had elevated β2 AR-AB levels. From this group, patients who planned IA treatment and fulfilled inclusion criteria were offered to participate in this trial and 23 were recruited for the study between September 2022 and October 2023. Three patients withdrew their consent to participate prior to the treatment due to concerns about the burden of study participation. 20 patients completed a six-month follow-up resulting in an 87% retention rate, suggesting that the protocol was generally acceptable to those who proceeded with the treatment.

The five IA treatment sessions were completed for all patients within a 10-day period in an outpatient setting. Sessions lasted between 4.5 and 9 h and were followed by a minimum of one day and a maximum of two days of rest. In five out of 20 patients, a Shaldon's catheter was required for vascular access; for the other patients peripheral venous puncture was sufficient. One patient experienced a thrombosis of the internal jugular vein as a side effect of the catheter. Otherwise, no severe side effects of IA treatment were reported.

The treatment was generally reported as physically exhausting and frequently triggered PEM. To mitigate this, we aimed to minimize external stressors, ensured proper oral hydration, and offered lorazepam for up to three days as supportive therapy.

The median disease duration was 22 months (IQR: 15–31) at the time of inclusion. Seven patients were male and 13 were female with a median age of 40 (IQR: 36–51). All patients had a severe degree of disability with a median Bell score of 30 (range 20–40), corresponding to reduction of the functional state to 50% and being usually housebound. Individual patient characteristics are displayed in Table 1.

**Table 1:** Cohort characteristics and response to treatment as assessed by the SF36 PF.

Patient	Gender (M/F)	Age (years)	Time since COVID-19 (months)	SF36 PF pre IA	SF36 PF post IA (M1)	ΔSF36 PF	Responder to IA (yes/no)
1	F	36	39	15	70	55	Yes
2	M	41	25	25	70	45	Yes
3	F	56	15	25	65	40	Yes
4	F	37	21	45	85	40	Yes
5	F	52	23	45	80	35	Yes
6	M	59	31	35	65	30	Yes
7	F	51	38	15	35	20	Yes
8	F	31	19	40	60	20	Yes
9	F	59	32	20	35	15	Yes
10	F	50	31	15	30	15	Yes
11	F	36	10	15	25	10	Yes
12	M	36	9	40	50	10	Yes
13	M	46	32	20	30	10	Yes
14	M	57	14	50	60	10	Yes
15	M	33	23	25	30	5	No
16	F	36	15	25	30	5	No
17	F	44	14	5	5	0	No
18	M	33	39	60	60	0	No
19	F	37	15	30	25	−5	No
20	F	39	19	50	45	−5	No

F: female; IA: immunoadsorption; M: male; M1: month 1 post immunoadsorption; SF36 PF: Short Form-36 physical functioning.

There was a positive correlation between the immune score, which depicts the severity of the lymph node pain, throat pain, and flu-like symptoms, and the levels of antibodies against β1 AR (*r* = 0.53, *p* = 0.017), β2 AR (*r* = 0.46, *p* = 0.040), M3 AChR (*r* = 0.60, *p* = 0.006), and M4 AChR (*r* = 0.60, *p* = 0.006) at baseline. Otherwise, no significant correlation was found between the autoantibody levels and the clinical presentation.

**Fig. 1:**
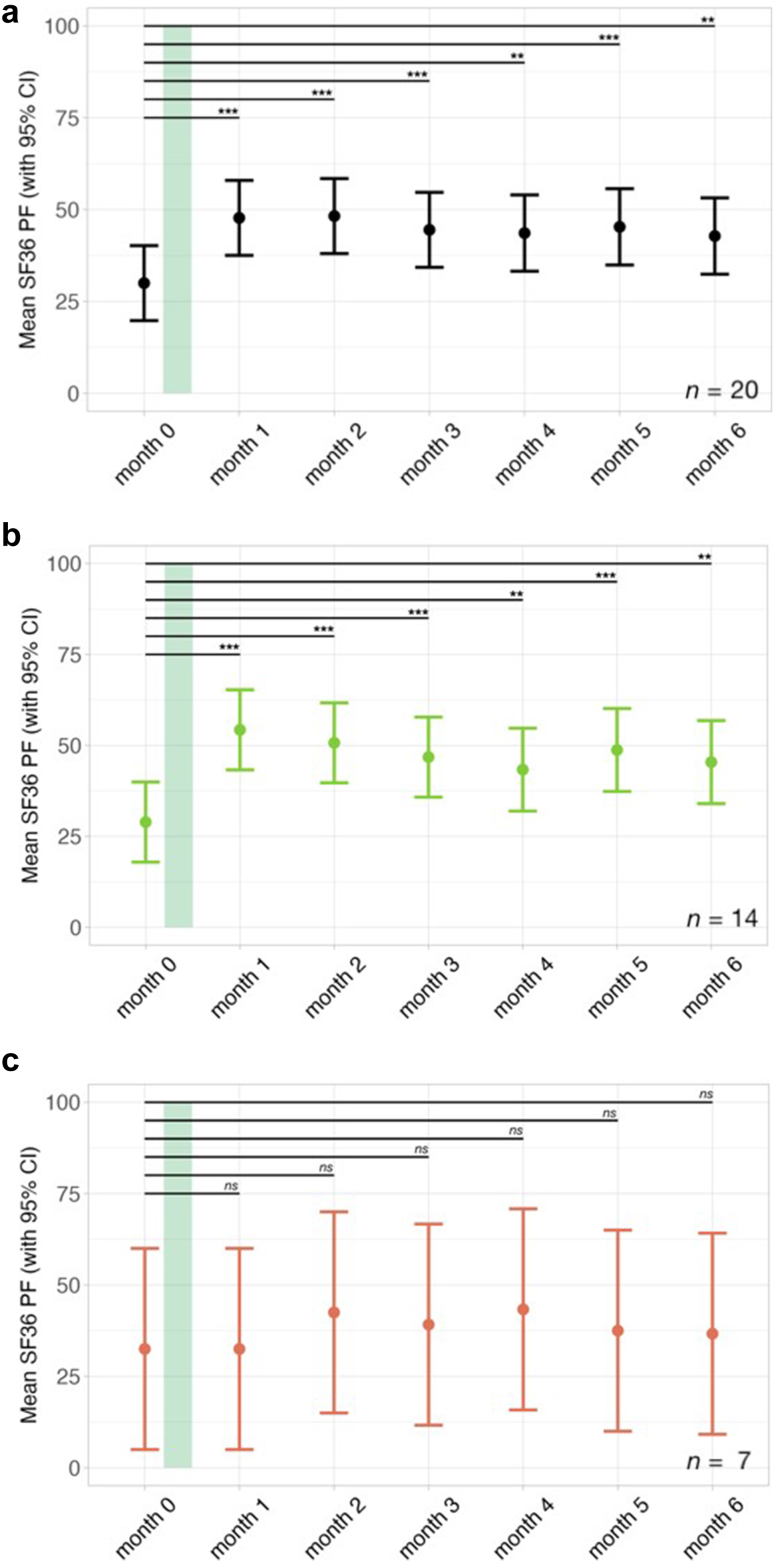
Course of the mean 36-Item Short-Form Survey physical functioning domain (SF36 PF) over the study period. The duration of IA therapy is indicated by the green bar. A higher score indicates less restriction in physical functioning. Error bars represent 95% confidence intervals. Data were analyzed using a linear mixed-effects model fitted by restricted maximum likelihood (REML), with t-tests computed using Satterthwaite's method for degrees of freedom with significance levels indicated as ∗*p* < 0.05, ∗∗*p* < 0.01, ∗∗∗*p* < 0.001. From top to bottom, the panels display the trajectories of the SF36 PF for: a) the whole cohort (*n* = 20), b) responders to the treatment defined by a ≥ ten-point increase in the SF36 PF at month 1 post IA (*n* = 14), and c) non-responders to the treatment defined by a ≤ ten-point increase in the SF36 PF at month 1 post IA (*n* = 7).

There was a mean increase in the SF-36 PF of 17.75 points (*CI*: 13.41–26.16, *p* < 0.001) four weeks post IA. The corresponding Cohen's d was calculated as 1.19, indicating a large effect size. There were, however, four patients with an increase of 10 points, which is considered as a clinically meaningful but small effect.^27^ As seen in Fig. 1, though the SF-36 PF scores tended to decrease between three and six months post IA, at month six post IA a significant mean improvement of 12.81 points (*CI*: 4.99–20.61, *p* = 0.002) remained. Fourteen out of 20 patients (70%) responded to the treatment as defined by an increase of at least 10 points in the SF-36 PF four weeks post IA suggesting a clinically meaningful improvement. Seven patients were non-responders according to this definition; however, two of these showed a delayed response at month 2.

There was no significant difference in age, symptom severity, disease duration, or level of β2 AR-AB between responders and non-responders. However, among female patients, responders had a significantly higher maximum HGS at baseline (*Mdn* = 23.5 kg, IQR: 17.7–25.5 kg) compared to non-responders (*Mdn* = 9.8 kg, IQR: 8.53–11.05 kg) (*z* = −2.62, *p* = 0.006, *r* = 0.73).

**Fig. 2:**
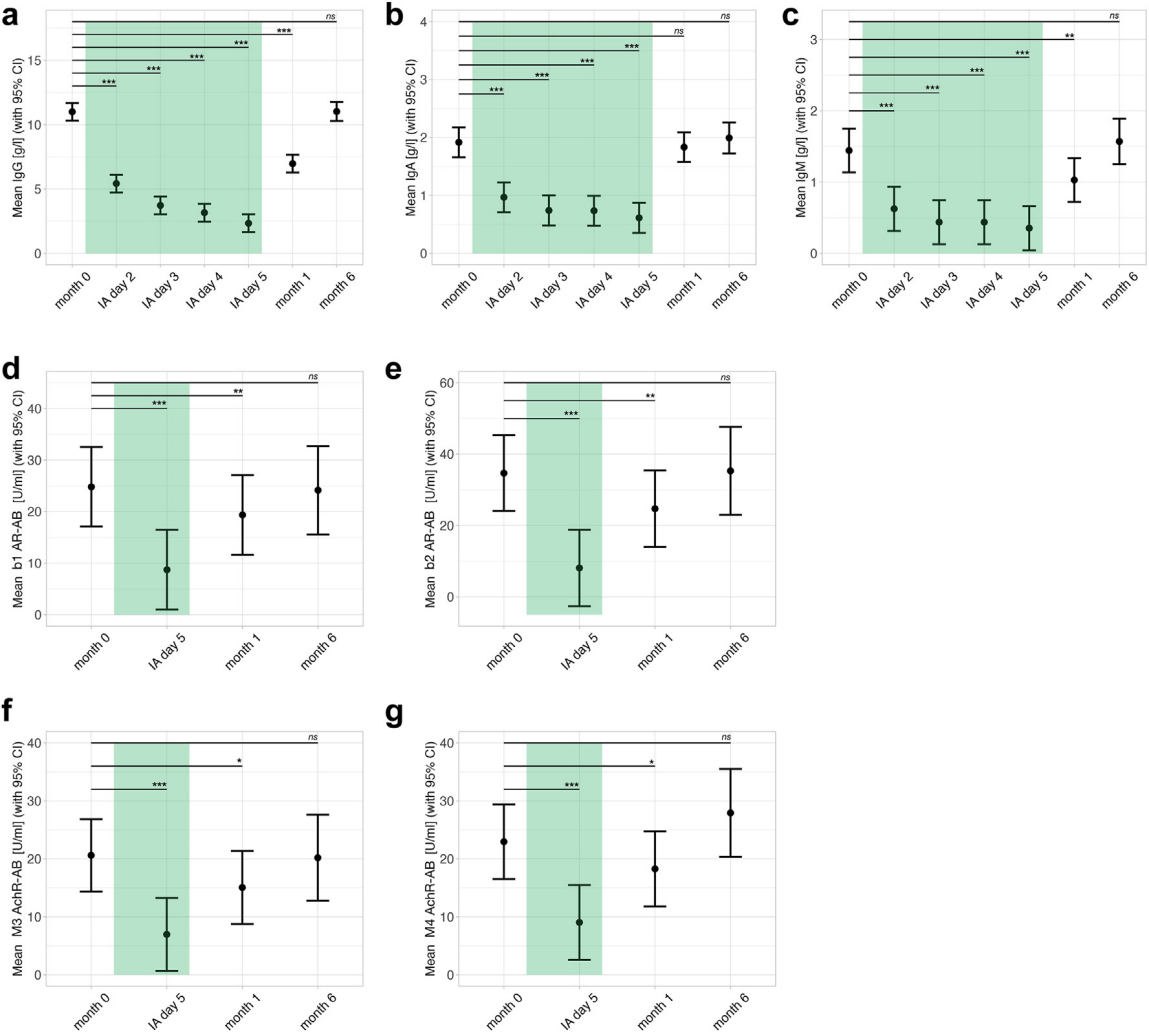
Course of immunoglobulin and autoantibody levels over the study period (*n* = 20), the duration of IA therapy is indicated by the green bar. Error bars represent 95% confidence intervals. Data were analyzed using a linear mixed-effects model fitted by restricted maximum likelihood (REML), with t-tests computed using Satterthwaite's method for degrees of freedom with significance levels indicated as ∗*p* < 0.05, ∗∗*p* < 0.01, ∗∗∗*p* < 0.001. From left to right, the panels display the trajectories of: a) immunoglobulin G (IgG), b) immunoglobulin A (IgA), c) immunoglobulin M (IgM), d) β1 adrenergic receptor autoantibodies (β1 AR-AB), e) β2 adrenergic receptor autoantibodies (β2 AR-AB), f) M3 acetylcholine receptor autoantibodies (M3 AchR-AB), and g) M4 acetylcholine receptor autoantibodies (M4 AchR-AB).

Serum IgG, IgA, and IgM levels were collected from all patients at baseline, before each treatment, and after treatment. Compared to baseline, IgG, IgA, and IgM were significantly decreased during IA treatment (*p* < 0.001) with a mean IgG reduction of 8.66 g/l (*CI*: 8.06–9.26 g/l) (79%, *CI*: 73–84%), mean IgA reduction of 1.3 g/l (*CI*: 1.2–1.51 g/l) (68%, *CI*: 63–78%), and mean IgM reduction of 1.1 g/l (*CI*: 0.84–1.34 d/l) (76%, *CI*: 58–93%) at day five of the treatment. Fig. 2a–c shows the course of immunoglobulin levels over time. β2 AR-AB decreased in parallel with the immunoglobulins with a mean reduction of 26.57 U/l (*CI*: 20.11–33.02 U/l) (77%, *CI*: 58–95%).

**Fig. 3:**
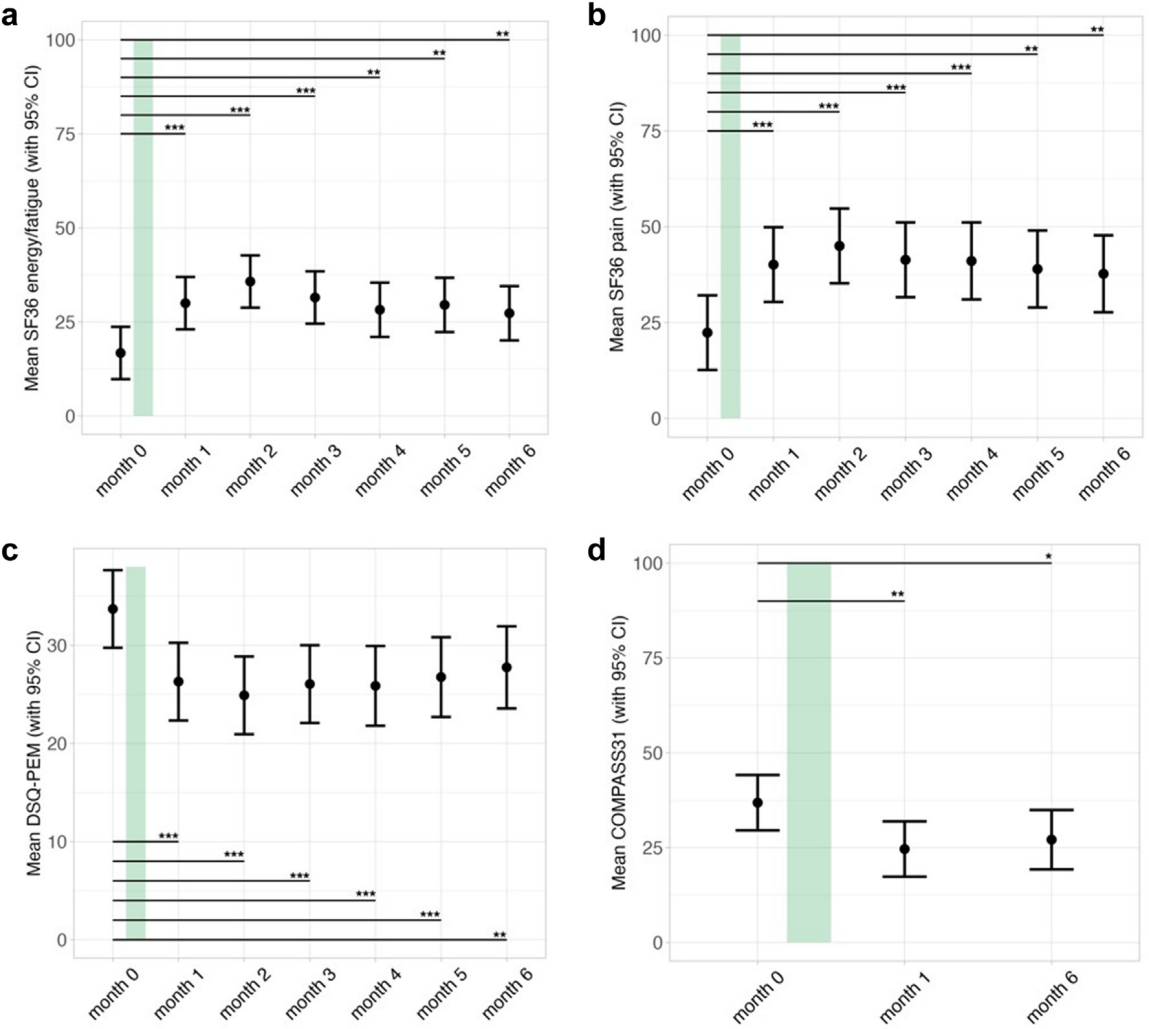
Clinical symptom progression over the study period (*n* = 20). The duration of IA therapy is indicated by the green bar. Error bars represent 95% confidence intervals. Data were analyzed using a linear mixed-effects model fitted by restricted maximum likelihood (REML), with t-tests computed using Satterthwaite's method for degrees of freedom with significance levels indicated as ∗*p* < 0.05, ∗∗*p* < 0.01, ∗∗∗*p* < 0.001. From left to right, the panels display the trajectories of: a) 36-Item Short-Form Survey energy/fatigue domain (SF36 energy/fatigue), where a higher score indicates less fatigue, b) 36-Item Short-Form Survey pain domain (SF36 pain), where a higher score indicates less pain, c) post-exertional malaise (PEM) as assessed by the DePaul Symptom Questionnaire (DSQ-PEM), higher scores indicate more severe PEM, and d) Composite Autonomic Symptom Score (COMPASS31), where a higher score indicates more autonomic symptoms.

Patients reported improvements in several key clinical symptoms at four weeks post IA. There were significant improvements in fatigue, as measured by the SF-36 energy/fatigue domain, and in pain, as measured by the SF-36 pain domain, following IA treatment. These improvements remained significant through month six. The maximal change was observed two months after IA, with a mean increase of 19 points (*CI*: 11.61–26.39, *p* < 0.001) and 22.63 points (*CI*: 13.28–31.96, *p* < 0.001) from baseline, respectively, as shown in Fig. 3.

Furthermore, patients reported a lasting improvement in autonomic symptoms, as shown in Fig. 3d. Improvements were most noticeable in the orthostatic, secretomotor, and gastrointestinal domains of the COMPASS31, which are shown in Figure S4 in the Supplement, as well as in the total score with a mean decrease of 12.23 points (*CI*: 19.06–5.4, *p* = 0.001) at month one.

All patients experienced severe PEM lasting at least 14 h, as specified by the inclusion criteria. Both frequency and severity of post-exertional symptoms significantly decreased after IA according to the DSQ-PEM. The total score is shown in Fig. 3c, remaining significantly decreased through month six.

Additionally, there was a significant mean decrease of 4.9 points (*CI*: 1.34–8.46, *p* = 0.009) in fatigue as assessed by the FSS at month one after IA as shown in Figure S3 in the Supplement. According to the symptom scores (NRS 1–10), there were also improvements in muscle pain, immunological as well as cognitive symptoms, that remained significant through month 6 with their maximum decrease between months two and three after IA as shown in Figure S1 in the Supplement.

The improvement in the degree of disability according to the Bell score post IA reached statistical significance only at months two, four, five, and six, with a maximum mean increase of 5.18 points (*CI*: 1.24–9.12, *p* = 0.013) at month two post IA as shown in Figure S2 in the Supplement.

**Fig. 4:**
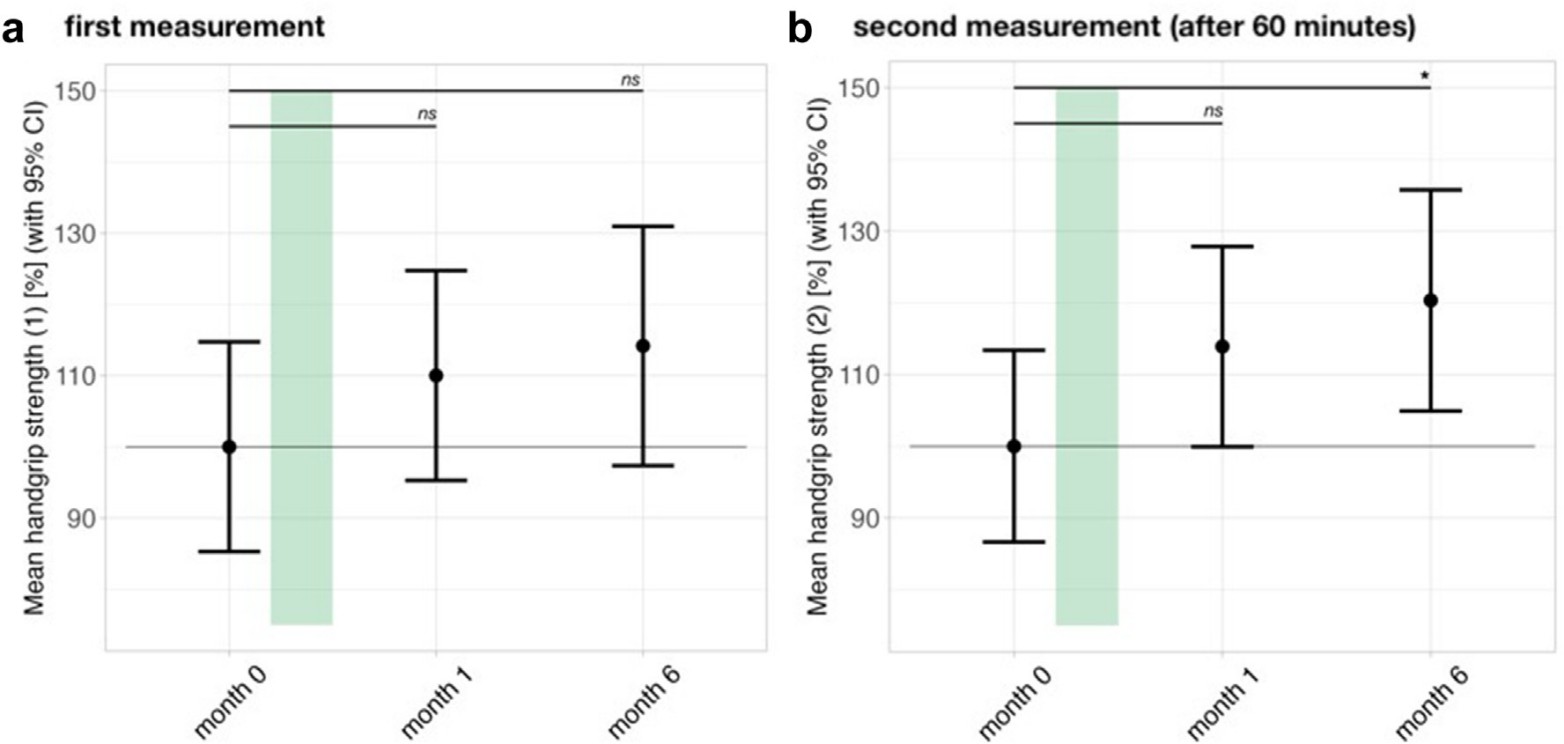
Course of mean handgrip strength (Fmean) in % in female patients (*n* = 13) measured a) initially and then b) repeated after one hour over the study period. The duration of IA therapy is indicated by the green bar. Error bars represent 95% confidence intervals. Data were analyzed using a linear mixed-effects model fitted by restricted maximum likelihood (REML), with t-tests computed using Satterthwaite's method for degrees of freedom with significance levels indicated as ∗*p* < 0.05, ∗∗*p* < 0.01, ∗∗∗*p* < 0.001.

To further quantify physical improvement, we measured repeat HGS as an objective marker of muscle fatigue. There were improvements, especially in repeat HGS before and after treatment in female patients as shown in Fig. 4. The mean HGS during the second measurement was significantly increased six months post IA by a mean of 20% (*CI*: 2–39%, *p* = 0.042) indicating a better recovery of strength within the 60 min of rest time between HGS measurements. In female responders (*n* = 9), both mean and maximum HGS were significantly increased as early as four weeks post-IA (not shown).

As a measure of endothelial dysfunction, the reactive hyperemia index (RHI) was assessed using an EndoPAT device. Six patients, three responders and three non-responders, had a reduced RHI (<1.68). There was no significant change in the RHI at six months post IA.

**Fig. 5:**
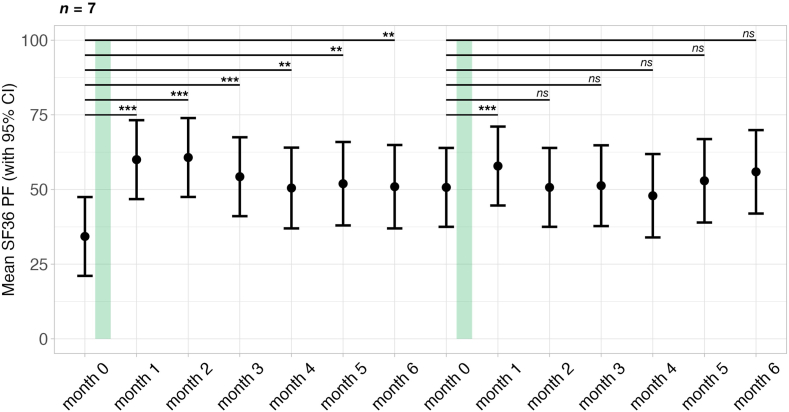
Course of 36-Item Short-Form Survey physical functioning domain (SF-36 PF) over the study period in patients who received a second cycle of IA therapy (*n* = 7). The duration of IA therapy is indicated by the green bar. Error bars represent 95% confidence intervals. Data were analyzed using a linear mixed-effects model fitted by restricted maximum likelihood (REML) for each cycle, with t-tests computed using Satterthwaite's method for degrees of freedom with significance levels indicated as ∗*p* < 0.05, ∗∗*p* < 0.01, ∗∗∗*p* < 0.001.

Responders to the first IA treatment were offered a second IA treatment within six to 12 months after completing the initial cycle, when their physical function or symptoms worsened again. The course of physical functioning according to the SF-36 PF in the seven patients receiving a repeat IA is shown in Fig. 5. Symptoms improved again four weeks after the second IA treatment but then remained at a similar level as after the first IA treatment.

## Discussion

There is increasing evidence that autoantibodies, including those targeting β-adrenergic and muscarinic acetylcholine receptors, may contribute to the pathophysiology of PCS and ME/CFS. We here provide evidence from an observational study that depletion of autoantibodies by IA can lead to improvement in overall physical functioning, as well as the severity of several key symptoms, including PEM, fatigue, pain, immunological, cognitive, and autonomic symptoms in a subset of post-COVID ME/CFS patients.

The clinical improvements observed after autoantibody removal via IA support the hypothesized involvement of these autoantibodies in the pathophysiology of ME/CFS and PCS in the responders. Meanwhile, a smaller group of patients did not show a response to the treatment. This highlights the potential diverse mechanisms underlying this condition, and indicates that autoantibodies may play a role only in a subgroup of patients.

IA has demonstrated clinical improvements in post-infectious ME/CFS patients in two small pre-pandemic trials conducted by our group.^19^^,^^20^ Although in most patients symptoms worsened after a few months, IA can induce longer lasting improvement for more than 12 months in a subset as observed in our previous study in postinfectious ME/CFS.^20^ In this study, clinical improvements generally peaked between months two and three, then gradually declined. However, even after six months, statistically significant improvements compared to baseline were still evident.

It is important to acknowledge that the observed improvement in SF36 physical functioning does not apply to all patients as there were several non-responders to the treatment and four patients had only a small improvement. Among these, some patients showed improvement only after a delay of two or three months, while others did not demonstrate any noticeable improvement. Thus, it is important to study the efficacy of autoantibody-depleting therapies in larger controlled trials. Further identifying factors predicting individual responses would support the clinical findings. To address this question, we are currently conducting several additional analyses to identify potential biomarkers for treatment response. These analyses include a comprehensive B cell subtyping using CyTOF analysis, the measurement of further autoantibodies, and markers of immune activation, hypoperfusion, microclots, and SARS-CoV-2 persistence. Although the main effect of IA is the depletion of autoantibodies, there is some evidence that memory B cells can be affected by IA.^19^ A potentially important finding is that responders had a higher baseline maximum HGS, suggesting they have less severe muscular or mitochondrial impairment.^35^ We could not identify any other significant differences in the clinical phenotypes of responders compared to non-responders. However, ME/CFS patients are a heterogeneous group, and there may be comorbid conditions, such as structural neuroanatomic abnormalities, that may have been overlooked in our patient cohort.

Growing evidence suggests that SARS-CoV-2 can trigger autoimmune processes, contributing to long-term effects of COVID-19. Dobrowolska et al. summarized recent findings indicating that PCS can develop autoantibodies against a range of antigens, including those specific to the immune and cardiovascular systems, the thyroid, and rheumatoid-specific targets, G-protein coupled receptors, and more. However, the clinical significance of most of these autoantibodies remains unclear.^36^ Several studies found elevated autoantibodies against β-adrenergic and muscarinic receptors in PCS patients and demonstrated an association between the levels of these autoantibodies with fatigue, neurological symptoms and pain.^9^, ^10^, ^11^, ^12^, ^13^^,^^37^^,^^38^ Importantly, two recent studies by Chen et al. as well as Santos Guedes de Sa et al. were able to induce similar symptoms in mice by transferring IgG from PCS patients.^39^^,^^40^ Remarkably, Chen et al. showed that patterns of transferred symptoms varied depending on the plasma proteome signature of the patients. IgG from those with neuronal or immune involvement induced pain, while IgG from those with muscular involvement impaired motion in the mice.^39^ In the study by Santos Guedes de Sa et al., patients showed a broad pattern of autoantibodies reactive with neuronal and endothelial tissues. IgG from individual patients induced distinct symptoms such as pain, hypersensitivity or loss of coordination in mice.^40^ These findings provide further evidence for the diversity of autoantibodies in PCS, possibly driven by prolonged inflammation, infection of various cell types and tissues, and reactivation of Epstein–Barr virus. Our IA study shows that removing autoantibodies generally led to disease improvement in a subset of patients; however, it remains unclear whether the benefits are primarily due to the removal of β2 AR-AB or other specific autoantibodies. Furthermore, decreases of β2 AR-AB and immunoglobulins were seen in all patients, independent of response.

The clinical improvement observed after autoantibody removal via IA in a majority of patients supports the hypothesized role of these autoantibodies in the pathophysiology of PCS and ME/CFS in the responders. Although IA has a rapid and high clinical efficacy in a subset of patients, the clinical benefits of IA are in some patients temporary, and the procedure is strenuous. During our trial, one case of internal jugular vein thrombosis occurred as a side effect of the catheter. Although the patient experienced no long-term effects following temporary anticoagulation, the risk of such complications should be considered. Furthermore, it is a highly specialized procedure, available only in select medical centers with the necessary equipment and expertise. B-cell and/or plasma cell depletion thus emerges as a promising treatment option, addressing the urgent need for more effective sustained therapeutic strategies. B cell depletion with the CD20 monoclonal antibody rituximab proved effective in a subset of ME/CFS patients, and its efficacy was found to be associated with the depletion of β2 AR-AB.^41^ However, the results from the phase II rituximab trials could not be confirmed in a multicenter trial, which had several limitations.^42^ We are currently initiating a trial with inebilizumab in ME/CFS and PCS patients, a monoclonal antibody directed against CD19 on B cells and plasma blasts showing high clinical efficacy in neuromyelitis optica.^43^ In this study, only responders to IA will be included in order to select patients with strong evidence of an autoantibody-mediated disease. Further novel treatment approaches are currently being tested in clinical trials for PCS and ME/CFS patients. These include targeting plasma cells by a monoclonal antibody to CD38, enhancing degradation of autoantibodies by Fc receptor inhibition, and neutralizing antibodies against G-protein coupled receptors with the aptamer BC007 (KTS-9-2022, NCT05633407, NCT05911009).

Limitations of this study include the small sample size and non-controlled study design. The study was designed to detect larger effect sizes, smaller effects may not be captured with this sample size. Further studies are therefore required; we and two other sites in Germany are currently conducting randomized controlled trials of IA in PCS and ME/CFS with larger sample sizes.^44^ Further, the outcomes are primarily patient-reported. The DSQ-PEM is designed for diagnostic purposes, and not validated for tracking changes in PEM in a clinical trial. While PCS encompasses a very heterogeneous patient population with presumably different pathomechanisms, our focus was solely on adult PCS patients meeting the ME/CFS criteria. Therefore, our findings may not be applicable to all PCS patients. Particularly our findings regarding the feasibility and safety of this procedure cannot directly be applied to the most severe, home bound patient group, since they were excluded from the study.

In conclusion, our study suggests that IA treatment may result in rapid clinical improvement in a subset of patients. Further trials with both more patients and clinical outcomes reported are needed to confirm our findings. Our study serves as proof of concept for the initiation of clinical trials using drugs specifically designed to target autoantibodies. Targeting B cells may offer a promising approach to long-term symptom relief by addressing the underlying mechanisms driving autoantibody generation.

## Contributors

ES conducted the main part of the study together with CK, KW, CS, and LK. RR helped with patient recruitment. CH prepared the figures of the report with input from LK and managed electronic data collection together with HF. CS did the conceptualization and provided resources and supervision. AK and MT managed IA therapy. FS managed laboratory analysis. LK wrote the first draft of the report and performed statistical analysis.

All authors had full access to all the data in the study and had final responsibility for the decision to submit for publication. CH and LK have accessed and verified the data.

## Data sharing statement

The data presented in this study will be available upon request from the corresponding author. Due to the sensitive nature of the data and the ongoing data collection and analysis, the data are not publicly available.

## Declaration of interests

Charité holds, together with CellTrend, a patent for the diagnostic use of autoantibodies against β2 AR-AB. CS has a consulting agreement with CellTrend and Berlin Cures. MT has received grants from the DFG, BMBF, Weidenhammer Zöbele Stiftung, Baxter, and Cytosorbents. MT has a consulting agreement with AstraZeneca and has received honoraria for lectures from Aey-Congress, AstraZeneca, Boehringer Ingelheim, Bayer, Baxter, Cytosorbents, DGK, DHL, Fresenius, Medical Tribune, MedPoint, Novartis, Pfizer, Sanofi, and Vifor. MT has also received support for attending meetings from AstraZeneca and Vifor. Additionally, MT serves on data safety monitoring or advisory boards for AstraZeneca, Boehringer Ingelheim, and Takeda. PG has received honoraria for lectures and travel support from Miltenyi Biotec GmbH. The other authors declare no conflict of interest.

## Appendix ASupplementary data

Synopsis Clinical Study Protocol
