# Supplementary material for: Efficacy of repeated immunoadsorption in patients with post-COVID myalgic encephalomyelitis/chronic fatigue syndrome and elevated β2-adrenergic receptor autoantibodies: a prospective cohort study
Source: Lancet Reg Health Eur. 2024 Dec 12;49:101161. doi: 10.1016/j.lanepe.2024.101161 (PMC11699797; doi:10.1016/j.lanepe.2024.101161)
Supplement: Synopsis Clinical Study Protocol [file mmc1.docx]

**Synopsis Clinical Study Protocol**

**Date/Version 9.11.22 Version 1.2 Blutentnahme Biomarker angepasst (**9.8.22, Version 1.1)

| **Titel** | Beobachtungsstudie zur Immunadsorption (IA) bei Patienten mit Autoantikörper-positivem Post-COVID-Syndrom (PCS)  Ethikvotum EA2_134_22 Charité vom 28.7.22 |
| --- | --- |
| **Hintergrund** | Nach einer milden bis moderaten ambulanten COVID-Infektion entwickeln etwa 10% ein Post-COVID-Syndrom (PCS) mit den häufigsten Symptomen Fatigue, Belastungsintoleranz, kognitiven Störungen, Kopf- und Muskelschmerzen und vielen weiteren Symptomen^1^. Das geht mit einer deutlichen Einschränkung der Lebensqualität einher, viele Betroffene können nur noch Teilzeit oder nicht mehr arbeiten^1^. Das PCS ist klinische ein sehr heterogenes Krankheitsbild, bei einem Teil der Patienten kann nach 6 Monaten ein ME/CFS diagnostiziert werden^1^. ME/CFS wird meistens durch einen Infekt ausgelöst und ist eine schwere, chronische Erkrankung^2^. Das Krankheitsbild ist gekennzeichnet durch eine schwere Fatigue mit dem Kernsymptom einer Belastungsintoleranz mit einer Zunahme der Beschwerden nach leichten Anstrengungen, der postexertionellen Malaise (PEM), die mindestens bis zum nächsten Tag anhält (> 14h). Weitere Symptome sind Kopf- und Muskelschmerzen, kognitive Einschränkungen (brain fog), orthostatische Intoleranz, autonome Funktionsstörungen, Schlafstörungen und ein schweres Krankheitsgefühl. Ein Teil der PCS Patienten, erfüllt die CCC Kriterien mit der Ausnahme, dass die PEM weniger als 14h anhält.  Es gibt bisher keine ursächliche Therapie. Die Therapieverfahren beschränken sich derzeit auf symptomorientierte und rehabilitative Interventionen. Ähnliches gilt für die Therapie des PCS. In Anbetracht der hohen und weiter steigenden Anzahl an PCS-Patienten und der Auswirkung des Krankheitsgeschehens auf das ohnehin stark belastete Gesundheitssystem und die Gesellschaft ist die Notwendigkeit der Entwicklung evidenzbasierter, standardisierter Therapieverfahren dringend geboten. Derzeit gibt es zahlreiche medikamentöse und nicht-medikamentöse Therapieansätze, die bereits von niedergelassenen Ärzten angewendet werden, für die es bisher keine ausreichende Evidenz durch kontrollierte Studien gibt.  Das PCS ist auch von den Mechanismen wahrscheinlich ein heterogenes Krankheitsbild. Es konnte gezeigt werden, dass COVID unterschiedliche Antikörper triggern kann. Bei einem Teil der PCS Patienten konnten Autoantikörper nachgewiesen werden, die auch mit dem Auftreten des PCS und der Symptomschwere korrelieren. Neben bekannten Autoantikörpern (Phospholipid, ANA, ENA) sind auch solche gegen Zytokine (IFNa, IL-2 u.a.), ApolipoproteinA, Adrenalin- und Acetylcholin-Rezeptoren nachgewiesen worden^3^.  Wir konnten in einer noch nicht veröffentlichten Studie beobachten, dass bei ME/CFS nach COVID, ähnlich wie bei ME/CSF nach anderen Infektionen, eine enge Korrelation der Adrenalin-Rezeptor Antikörper mit Symptomschwere besteht.  Die IA wird bei vielen Autoantikörper-vermittelten Erkrankungen erfolgreich eingesetzt. Es gibt eine Vielzahl von publizierten Studien bei unterschiedlichen Erkrankungen. Zwei Studien zum erfolgreichen Einsatz von IA bei ME/CSF wurden von unserem Institut im März 2018 und im Juli 2020 veröffentlicht. Bei ME/CFS nach anderen Infektionen haben wir bereits zwei kleine proof of concept Studien mit IA durchgeführt (EA2_063_15), die bei den meisten Patienten zur Besserung führte^4,5^. Eine IA ist auch bei vielen anderen Antikörper-vermittelten Autoimmun-erkrankungen wirksam.   1. C Kedor, H Freitag, L MeyerArndt, K Wittke, T Zoller, F Steinbeis, M Haffke, G Rudolf, B Heidecker,HD Volk, C Skurk, F Paul, J BellmannStrobl, Scheibenbogen. Post COVID-19 Syndrome and Chronic Fatigue Syndrome (ME/CFS) following the first pandemic wave in Germany – a first analysis of a prospective observational study. Nat Commun. 2022, im Druck 2. Scheibenbogen C, Kedor C, Behrends U. ME/CFS, Der niedergelassene Arzt, 12/2020 3. Rojas M, Rodríguez Y, Acosta-Ampudia Y, Monsalve DM, Zhu C, Li QZ, Ramírez-Santana C, Anaya JM. Autoimmunity is a hallmark of post-COVID syndrome. J Transl Med. 20:129, 2022 4. Scheibenbogen C, Loebel M, Freitag H, Krueger A, Bauer S, Antelmann M, Doehner W, Scherbakov N, Heidecke H, Reinke P, Volk HD, Grabowski P. [Immunoadsorption to remove ß2 adrenergic receptor antibodies in Chronic Fatigue Syndrome CFS/ME.](https://www.ncbi.nlm.nih.gov/pubmed/29543914) PLoS One. e0193672; 2018 5. Tölle M, Freitag H, Antelmann M, Hartwig J, Schuchardt M, van der Giet M, Eckardt KU, Grabowski P, Scheibenbogen C. Myalgic Encephalomyelitis/Chronic Fatigue Syndrome: Efficacy of Repeat Immunoadsorption. J Clin Med. 9(8):2443, 2020 |
| **Studienleiter** | Prof. Dr. Carmen Scheibenbogen  Institute of Medical Immunology  Charité – Universitätsmedizin Berlin  Augustenburger Platz 1  13353 Berlin  Tel: 030 – 450-524103  Fax: 030 – 450-7-524103  e-mail: [carmen.scheibenbogen@charite.de](mailto:carmen.scheibenbogen@charite.de)  PD Dr. Markus Tölle  Schwerpunkt Nephrologie und Intensivmedizin  Charité – Universitätsmedizin Berlin  Hindenburgdamm 30  12203 Berlin  e-mail: [markus.toelle@charite.de](mailto:markus.toelle@charite.de) |
| **Studientyp** | Beobachtungsstudie - Investigator initiated Non-Interventional trial |
| **Studiencenter** | Berlin Charité |
| **Patientenzahl** | 20 PCS Patienten, davon mindestens 10 Pat mit Diagnose ME/CFS nach CCC-Kriterien. Patienten, die die CCC-Kriterien nicht erfüllen wegen PEM weniger als 14 Stunden können ebenfalls aufgenommen werden, wenn die SEID-Kriterien erfüllt sind. |
| **Studiendauer** | Enrolment start date (FPI): 1.9.2022 |
|  | Enrolment finish date (LPI): 31.12.2022 |
|  | Treatment period end date (LP off treatment): 30.6.2023 |
|  | Follow-up period end date (LP off study): 30.6.2024 |
| **Studienziel** | Beobachtung und Dokumentation des Symptomverlaufs bei 20 Patienten mit PCS, die die Diagnosekriterien für ME/CFS erfüllen und bei denen eine Antikörperdepletion mittels IA durchgeführt wird.  Die Studie wird als eine nicht-interventionelle Beobachtungsstudie durchgeführt. Eine IA mit der TheraSorb®-Säule von Miltenyi bei long/Post-COVID-Syndrom erfolgt in der zugelassenen Anwendung.  Hypothese:  Die Antikörperdepletion mittels IA führt zur Symptomverbesserung bei Patienten mit Autoantikörper-positivem PCS. |
| **Primäre und sekundäre Ziele** | Primäre Studienziel |
|  | Nachweis einer Symptomverbesserung für mind. 4 Wochen nach der IA, erfasst durch den SF 36- Körperliche Funktion (PF) Fragebogen. Der SF-36 PF wurde am häufigsten als primärer Endpunkt in klinischen Studien zu ME/CFS verwendet. Es wurde gezeigt, dass ein Anstieg von mindestens 10 Punkten im SF-36-PF (Bereich 0 - 100 = gesund) eine klinisch relevante Verbesserung ("ein wenig besser") und ein Anstieg von 20 Punkten eine größere klinische Verbesserung ("viel besser") definiert (Brigden 2018). Daher wird ein Anstieg von mindestens 10 Punkten über mindestens 4 Wochen als Ansprechen definiert und als primärer Endpunkt bewertet.  Sekundäre Studienziele sind:   - Verbesserung der Arbeitsfähigkeit - Verbesserung der Handkraft - Verbesserung der Gefäßfunktion: Endotheliale Dysfunktion (Endopat) und orthostatische Intoleranz (Puls und Blutdruck im Sitzen und Stehen) - Verbesserung der Werte in der Chalder Fatigue Skala, CCC Symptomscore, MBSQ und COMPASS-31 - Abfall der Autoantikörper und löslichen Biomarker - Beobachtung der Verträglichkeit |
| **Studienablauf** | Patienten, die sich in unserer Post Covid Ambulanz vorgestellt haben, die die kanadischen Kriterien (CCC) für eine ME/CFS erfüllen (PEM über mindestens 14h oder länger) und bei denen Autoantikörper nachgewiesen werden, wird die Möglichkeit einer IA sowie die Teilnahme an der Beobachtungsstudie angeboten. Auch PCS Patienten, die die CCC Kriterien erfüllen wegen einer kürzeren PEM (< 14h) aber die SEID-Kriterien erfüllen und eine IA bekommen, können in die Studie aufgenommen werden.  Die IA erfolgt studienunabhängig an 5 Tagen ambulant in der Klinik für Nephrologie CBF/CVK oder CCM (unter Leitung von PD Dr. Markus Tölle). Tage der IA: 1-2-4-6-8, aber auch Abweichungen sind möglich in einem Gesamtzeitraum von max 2 Wochen mit dem Ziel der Reduktion des IgG um etwa 90% nach 5 Tagen. Nach der 5. IA werden bei Bedarf 10g IgG substituiert. Die IA soll bei Ansprechen nach 3 und 6 Monaten wiederholt werden. Dieses Vorgehen entspricht der klinischen Routine und ist von der Studienteilnahme unabhängig.  Bei allen Patienten erfolgt zum Zeitpunkt des Studieneinschluss die Bestimmung von Basislabor, Immunglobulinen, Autoantikörpern und Biomarkern für PCS (Zytokine, Endothelin) mit gesamt 30 ml Blutentnahme. Die Diagnose ME/CFS wird mit Hilfe der Kanadischen Kriterien gestellt. Mit Fragebögen erfolgt die Erfassung des Gesundheitszustandes (SF36), der Arbeitsfähigkeit in den letzten 12 Monaten, der Schwere der Symptome (Kanad. Kriterien gewichtet, Chalder Fatigue Questionnaire, MBSQ, Bell Score). Diese werden im Verlauf wöchentlich im ersten Monat, dann alle 4 Wochen über 12 Monate nach Therapieende erhoben. Da für das Ausfüllen der Fragebögen bis zu einer Stunde Zeit kalkuliert wird, können die Patienten diese zu Hause ausfüllen.  Folgende Diagnostik wird vor Beginn der IA und alle 3 Monate bis 12 Monate nach Therapieende durchgeführt:   - Endopat Test zur nicht invasiven Messung der endothelialien Dysfunktion. Das Endo-PAT2000 ist eine nicht-invasive Untersuchungsmethode zur Messung der Gefäßfunktion. Dabei wird ein Biosensor auf den Zeigerfinger beider Arme aufgesetzt. Nach Anlage einer Blutdruckmanschette am Oberarm wird die Pulswellenamplitude der Fingerarterien vor bzw. nach der venösen Stauung (Inflation der Manschette mit 20 mmHg über den systolischen Blutdruck) gemessen. Die Aufzeichnung der Pulswellen am anderen Arm dient als Kontrolle. Die Messung erfolgt liegend und dauert etwa 15 min. Diese Untersuchung wird in Zusammenarbeit mit der Medizinischen Klinik m. S. Kardiologie und der Arbeitsgruppe „Interdisziplinäre Schlaganfallforschung“ durchgeführt. Handkraftmessung, Messung der Handkraft mit Hilfe eines Handkraftmessgerätes erfolgt mit der dominierenden Hand (Rechtshänder= rechte Hand, Linkshänder = linke Hand). Nachdem der Patient eine Einweisung über die Durchführung der Messung erhalten hat, erhält  der Patient den eingestellten Handkraftmesser in die Hand zieht für 3 Sekunden so kräftig wie möglich daran. Diese Übungen werden 10 Mal durchgeführt mit je 5 Sekunden Pause dazwischen und nach 1 Stunde wiederholt. - NASA lean (Anlehn) Test zur Erfassung des orthostatischen Puls und Blutdruck (Dauer: 15 min), dabei wird die Herzfrequenz und der Blutdruck nach 5 Minuten Liegezeit und nach dem Aufstehen und an der Wand gelehnt stehen (10x jede Minute, insgesamt 10 Minuten) gemessen. - Blutabnahme zur Bestimmung von Routinelabor, IgG/A/M, Autoantikörpern und Biomarkern (Zytokine, Endothelin) (zusätzlich 20 ml, die Entnahme soll nach Möglichkeit im Rahmen der routinemäßigen Blutentnahme erfolgen)   Vor und nach jeder IA werden IgG/A/M, Autoantikörper und lösliche Marker gemessen.  Die Studiendauer beträgt 12 Monate nach der IA, bei Patienten, die weitere IA an Monat 3 und 6 erhalten also 18 Monate. |
| **Einschlusskriterien:** | - einwilligungsfähige Patienten im Alter von 18 – 65 Jahren mit PCS mindestens 6 Monate nach COVID-19 und:   - ME/CFS diagnostiziert nach den Kanadischen Konsensuskriterien (CCC) mit Belastungsintoleranz und Symptomverschlechterung über mind. 14 Stunden (ME/CFS) oder   - Kanadische Konsensuskriterien (CCC) nicht vollständig erfüllt, da die Belastungsintoleranz zu einer Symptomverschlechterung führt, die weniger als 14 Stunden anhält (diagnostisch als Post Covid Syndrom (PCS) eingeordnet, wie in Ref. 1 beschrieben)   - Relevante Beeinträchtigung durch die Erkrankung mit Bell-Score < 60 - Nachweis einer COVID-Infektion zu Erkrankungsbeginn (PCR) oder N-IgG - Nachweis von Autoantikörpern (adrenerge Antikörper, ANA, antineuronale Antikörper u.w) - Immunadsorption mit der TheraSorb®-Säule für 5 Tage - Einwilligung durch den Patienten - Krankenversicherung |
| **Ausschlusskriterien:** | - fehlende Bereitschaft zur Speicherung pseudonymisierter Krankheitsdaten im Rahmen der Studie - Schwangerschaft - andere Erkrankungen, die die Diagnose PCS nicht sicher stellen lassen (z.B. Herz-, Lungenerkrankung, schwere Depression, Krebserkrankung) - Organerkrankungen im Rahmen des PCS (u.a. definierte Autoimmunerkrankung, ILD, Myokarditis) - akute Infektion (COVID, HIV, Hepatitis u.a.) - schwere ME/CFS Erkrankung mit Bettlägerigkeit |
| **Therapieschema** | **Produkt:** Therasorb, Miltenyi  **Dosing schedule:**  Die Behandlung in dieser Studie besteht aus 3 IA-Zyklen mit 5 Behandlungstagen (IA-Tage: 1,2 - 4 - 6 - 8). Andere Tage sind möglich, aber die IA sollte innerhalb von maximal 2 Wochen durchgeführt werden. 10 g IgG werden nach der 5. IA optional ersetzt.  Die Behandlung kann jederzeit abgebrochen werden, wenn die Patienten inakzeptable Toxizitäten entwickeln. |
| **Studiendauer pro Patient** | Die geplante Studiendauer pro Patient ist 12 - 18 Monate (1- 6 Monate Behandlung, 12 Monate Nachbeobachtung)  Patienten, die eine Symptombesserung nach der 1. IA haben, erhalten zwei weitere IA nach 3 und 6 Monaten. |
| **Tolerability/safety** | AE und SAE werden berichtet. |
| **Statistical methods** | deskriptiv |
| **Studienende** | Das Ende der Studie ist definiert als die letzte Nachuntersuchung (12 Monate nach Ende der Behandlung) des letzten Patienten. |
| **Studienvisiten**  **Fragebögen**  **Biomarker** | siehe Tabelle |

| **Procedure** | **Screening**  **(up to 28 days before V2)*** | **Immunoadsorption**  **For each cycle 1 – 3: V2+V3, Q1-3** | | | | | | **Follow-up**  **(6 and 12 month after last IA)** |
| --- | --- | --- | --- | --- | --- | --- | --- | --- |
|  |  | Days -7 - -1 | Before 1^st^ IA | During IA  Days 1 - 5 | Weeks 1 - 4 | Week 4 | Months  2 – 12 (-18) | ±2 weeks |
| **Visits (V)**  **Questionnaires (Q)** | **V1** | **V2** | **Qu1** | **Qu1** | **Qu3** | **V3** | **Qu4** | **V4+5** |
| Informed consent | X |  |  |  |  |  |  |  |
| Inclusion/exclusion criteria | X |  |  |  |  |  |  |  |
| Demography | X |  |  |  |  |  |  |  |
| Physical examination | X |  |  |  |  |  |  |  |
| Medical history, work ability last 6/12 months | X |  |  |  |  |  |  | X |
| Current medical conditions | X |  |  |  |  |  |  | X |
| Concomitant medication | X | X |  |  |  | X | X | X |
| AE/SAE review |  | ====== | | | | | | |
| **Questionnaires** |  |  |  |  |  |  |  |  |
| SF-36 Health Status | X | X |  |  |  | X | X | X |
| Bell Disability Scale (Bell) | X | X |  |  |  | X | X | X |
| Self-reported Symptoms (MBSQ+MBLCSQ) | X |  |  |  |  | X |  | X |
| PEM criteria | X |  |  |  |  | X | X | X |
| Fatigue severity scale | X | X |  |  |  | X | X | X |
| CCC Symptom Score | X | X | X | X | X | X | X | X |
| COMPASS-31 | X |  |  |  |  | X |  | X |
| PHQ4 | X |  |  |  |  |  |  |  |
| **Functional tests** |  |  |  |  |  |  |  |  |
| SDMT (cognitive test) | X |  |  |  |  | X |  | X |
| EndoPAT Test (Itamar) | X |  |  |  |  |  |  | X |
| Handgrip-Muscle-Fatigue Test (HGS) | X | X |  |  |  | X |  | X |
| Passive Standing Test (NIH CDE) | X | X |  |  |  | X |  | X |
| **Laboratory parameter**  **as part of routine diagnostics** |  |  |  |  |  |  |  |  |
| Ferritin | X |  |  |  |  |  |  |  |
| Folic acid | X |  |  |  |  |  |  |  |
| TSH | X |  |  |  |  |  |  |  |
| ANA, ENA-Screen, if positive all ENA antibodies | X |  |  |  |  |  |  |  |
| IgG, IgA, IgM, IgE | X |  |  |  |  | X |  | X |
| IgG 1, 2, 3, 4 Se | X |  |  |  |  | X |  | X |
| CBC, hematocrit, albumin | X | X |  |  |  | X |  | X |
| Creatinine | X | X |  |  |  | X |  | X |
| Na, K, Ca, phosphate | X | X |  |  |  | x |  | x |
| Plasma total protein | X |  |  |  |  |  |  |  |
| Quick, PTT, fibriniogen | X |  |  |  |  |  |  |  |
| CRP | X | **X** |  |  |  | X |  | X |
| IL-8 (after erythrocyte lysis) | X |  |  |  |  | X |  | X |
| GPT/ALT, GOT/AST, gamma GT, AP, LDH | X |  |  |  |  | X |  | X |
| IgG/A/M zusätzlich |  |  |  | **X (täglich)** |  |  |  |  |
| **Biomarker**^$^ |  |  |  |  |  |  |  |  |
| ß2R + other GPCR autoantibodies**^1^** |  | X^$^ |  | **X ^$^ 5. IA**  **(direkt vor 5. IA)** |  | X ^$^ |  | X ^$^ |
| Proteomics^2^ |  | X^$^ |  | X ^$^ |  |  |  |  |
| Single cell RNA sequencing (SCS)^2^ |  | X^$^ |  | X ^$^ |  |  |  |  |
| Cytokines and further soluble markers to confirm proteomics and SCS^2^ |  | X ^$^ |  | X ^$^ |  |  |  |  |
| Flow cytometry for immune cell phenotyping^2^ |  | X ^$^ |  | X ^$^ |  |  |  |  |

1 Serum 10ml Labor Scheibenbogen

2 Heparin 3x9ml, EDTA 1x9 ml, Serum 1x8ml Labor Sawitzki
