# Supplementary material for: Efficacy of repeated immunoadsorption in patients with post-COVID myalgic encephalomyelitis/chronic fatigue syndrome and elevated β2-adrenergic receptor autoantibodies: a prospective cohort study
Source: Lancet Reg Health Eur. 2024 Dec 12;49:101161. doi: 10.1016/j.lanepe.2024.101161 (PMC11699797; doi:10.1016/j.lanepe.2024.101161)
Supplement: Figures S1–S4 [file mmc2.docx]

**Supplementary Material - Efficacy of Repeat Immunoadsorption in Post-COVID ME/CFS Patients with Elevated β2-Adrenergic Receptor Autoantibodies: a Prospective Cohort Study**


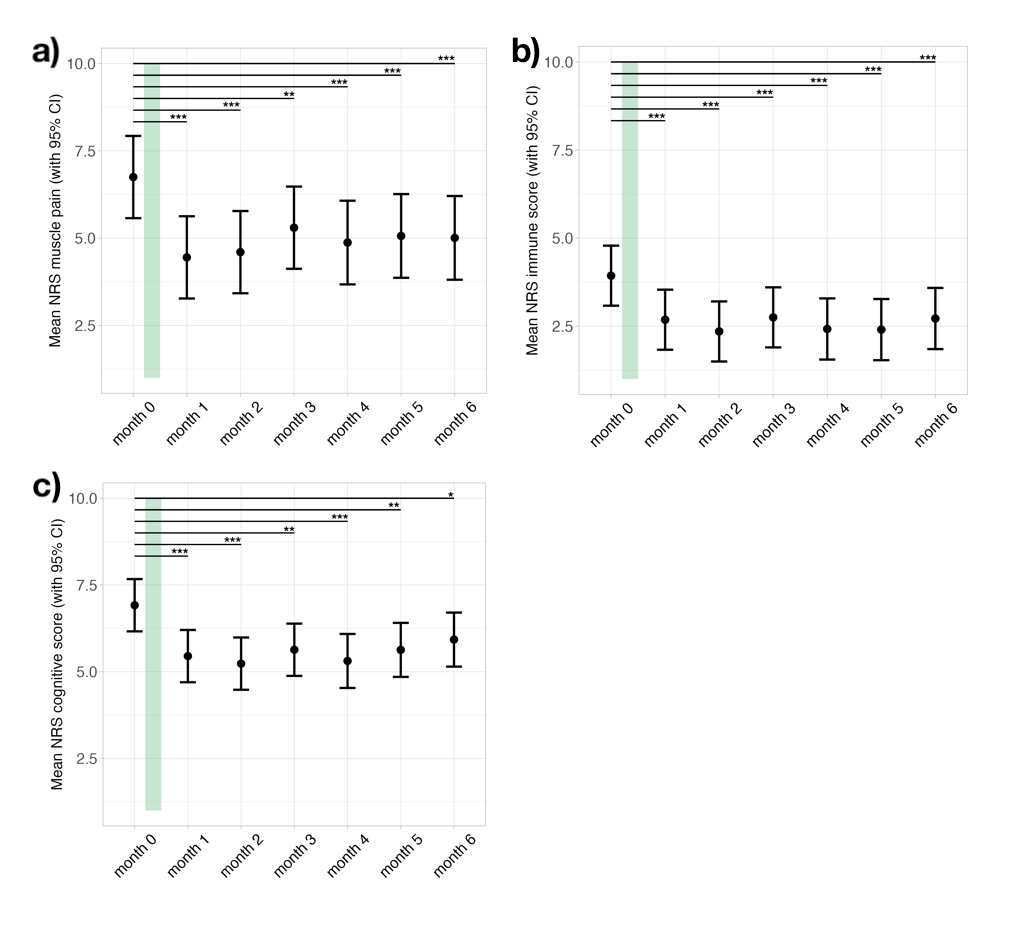


Figure S1: Clinical symptom progression as assessed by a numeric rating scale (NRS) from 1 to 10, with 10 indicating maximum symptom severity, over the study period (*n*=20). Error bars represent 95% confidence intervals. Data were analyzed using a linear mixed-effects model fitted by restricted maximum likelihood (REML), with t-tests computed using Satterthwaite's method for degrees of freedom with significance levels indicated as * *p* < 0·05, ** *p* < 0·01, *** *p* < 0·001. From left to right, the panels display the trajectories of: a) muscle pain b) immunological dysfunction (mean of lymph node pain, throat pain, and flu-like feeling) c) cognitive dysfunction (mean of memory disturbance, concentration problems, and mental tiredness)


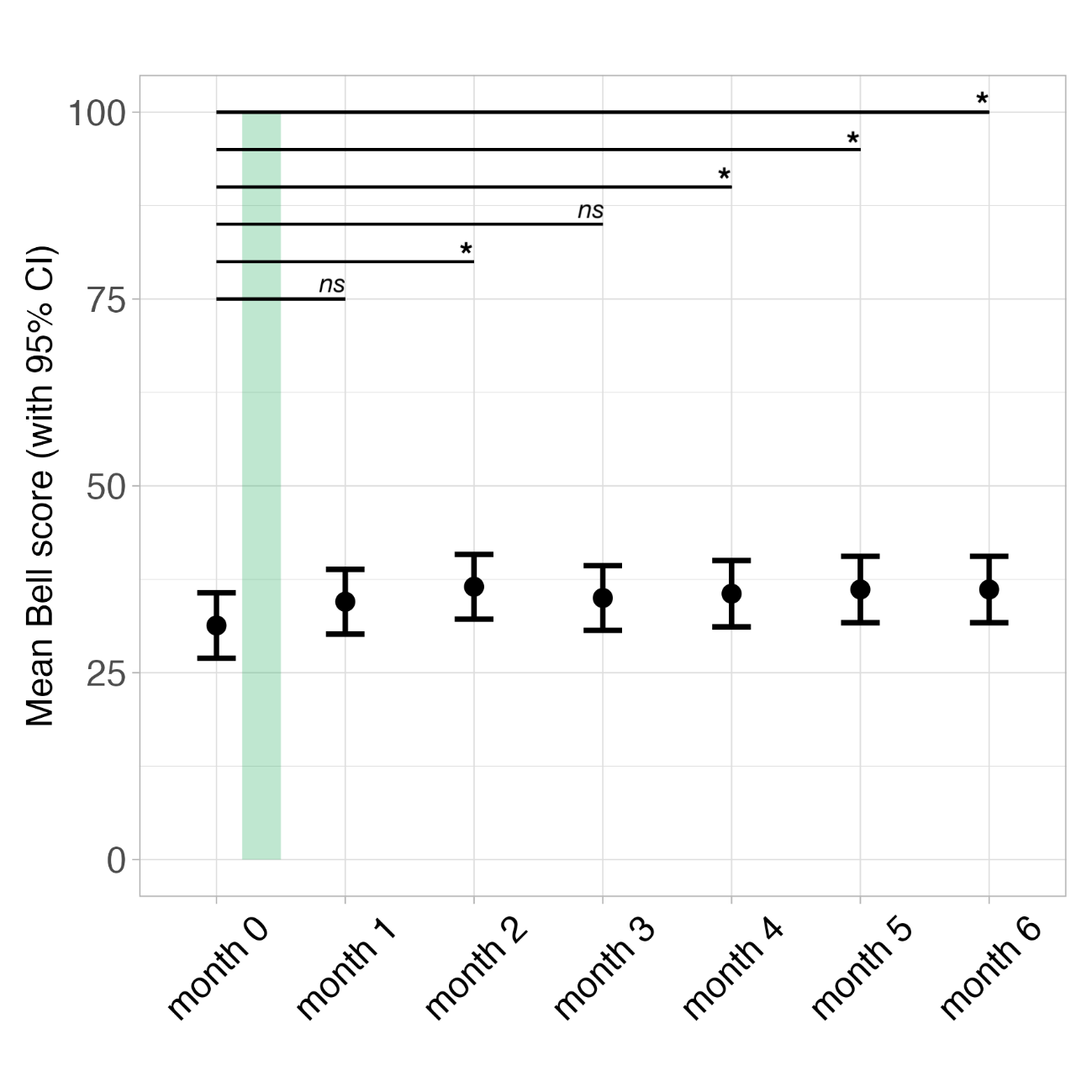


Figure S2: Disease severity as assessed by the Bell Score, with 100 indicating maximum disease severity, over the study period (*n*=20). Error bars represent 95% confidence intervals. Data were analyzed using a linear mixed-effects model fitted by restricted maximum likelihood (REML), with t-tests computed using Satterthwaite's method for degrees of freedom with significance levels indicated as * *p* < 0·05, ** *p* < 0·01, *** *p* < 0·001.


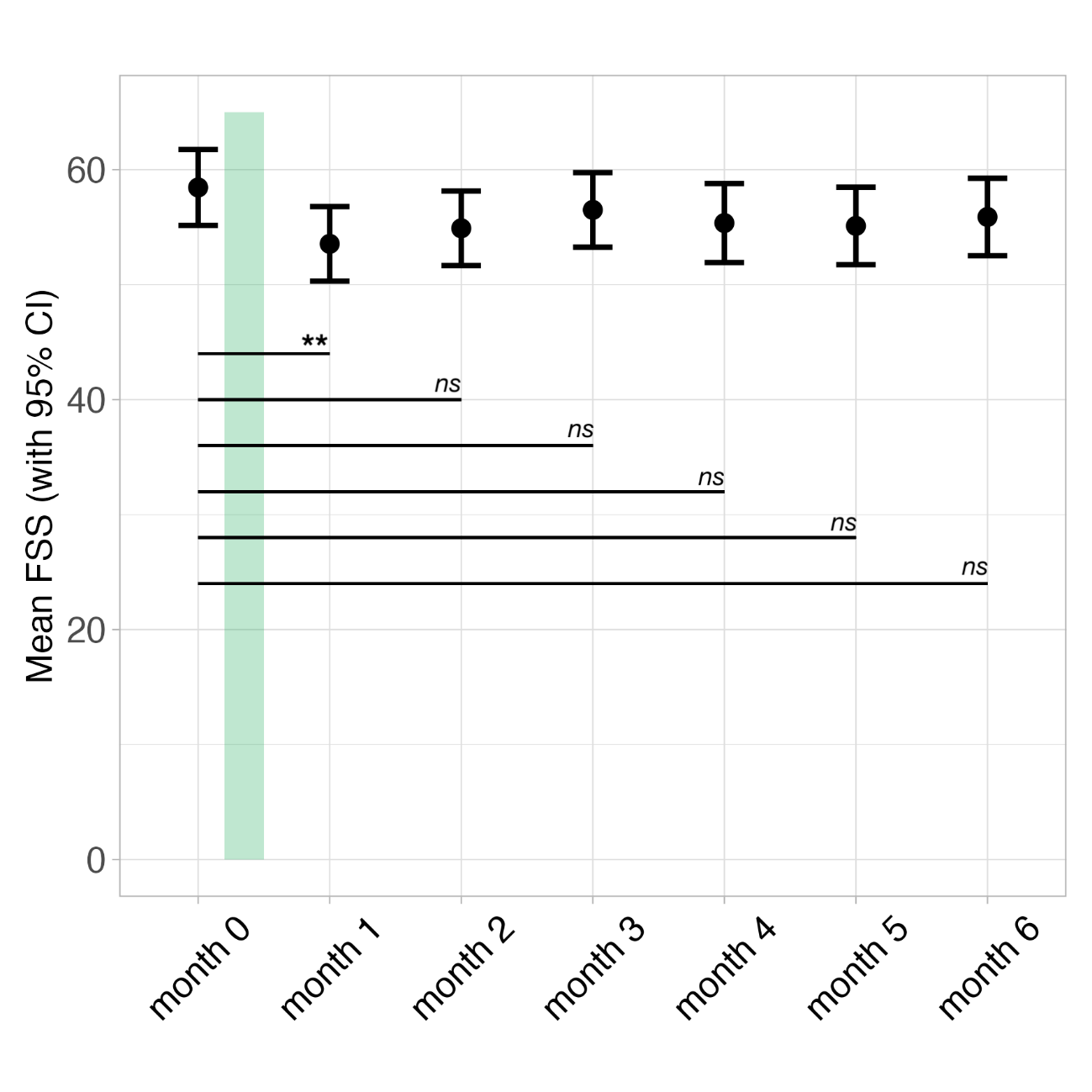


Figure S3: Fatigue as assessed by the Fatigue Severity Scale (FSS), with higher values indicating more severe fatigue, over the study period (*n*=20). Error bars represent 95% confidence intervals. Data were analyzed using a linear mixed-effects model fitted by restricted maximum likelihood (REML), with t-tests computed using Satterthwaite's method for degrees of freedom with significance levels indicated as * *p* < 0·05, ** *p* < 0·01, *** *p* < 0·001.


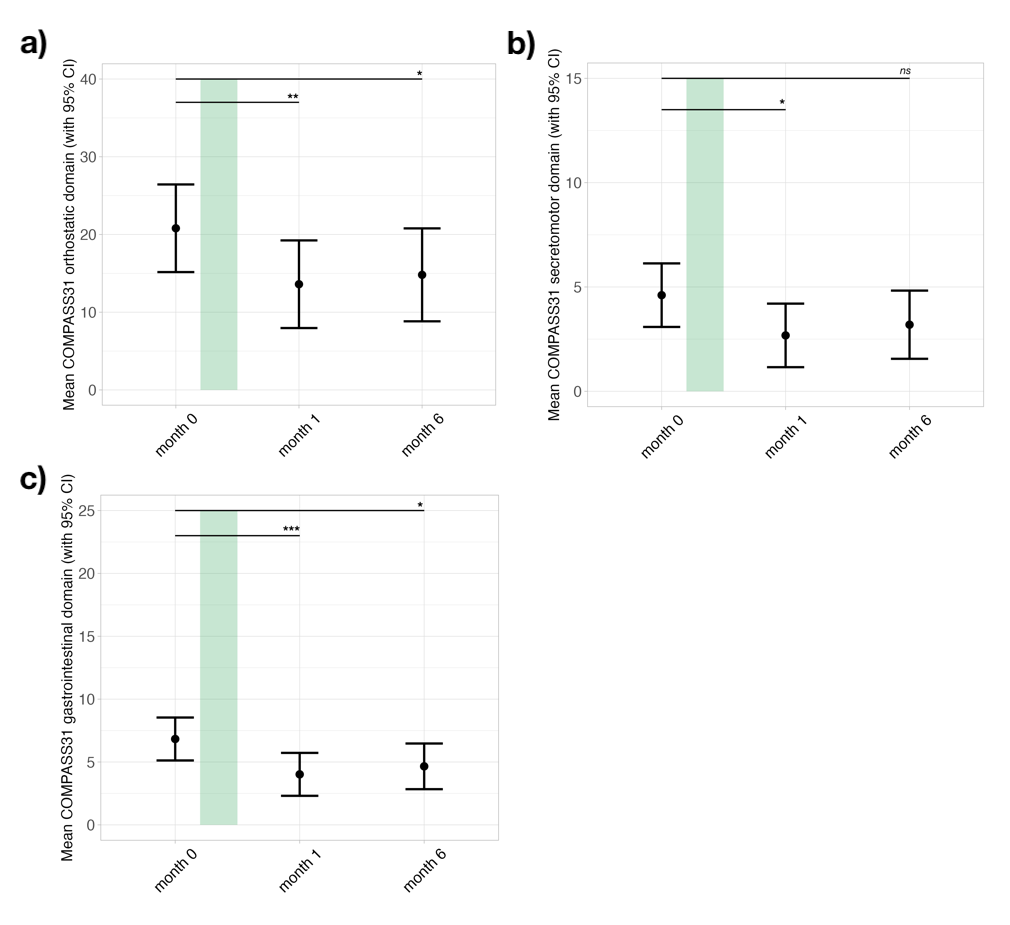


Figure S4: Autonomic symptoms as assessed by the Composite Autonomic Symptom Score (COMPASS31), where a higher score indicates more autonomic symptoms, over the study period (*n*=20). Error bars represent 95% confidence intervals. Data were analyzed using a linear mixed-effects model fitted by restricted maximum likelihood (REML), with t-tests computed using Satterthwaite's method for degrees of freedom with significance levels indicated as * *p* < 0·05, ** *p* < 0·01, *** *p* < 0·001. From left to right, the panels display the trajectories of the: a) orthostatic intolerance domain b) secretomotor domain c) gastrointestinal domain
